# How to Evaluate and Adjust the Recommended Level of Physical Activity in Patients with Congenital Heart Diseases? A Practical Approach

**DOI:** 10.3390/jcm14228126

**Published:** 2025-11-17

**Authors:** Dominika Filipiak-Strzecka, Ibadete Bytyçi, Agata Bielecka-Dabrowa

**Affiliations:** 1 Department of Cardiology and Congenital Heart Diseases of Adults, Polish Mother’s Memorial Hospital Research Institute (PMMHRI), 93-338 Lodz, Poland; dominika.filipiak-strzecka@iczmp.edu.pl; 2 Medical Faculty, University of Prishtina, 10000 Prishtina, Kosovo; 3 Clinic of Cardiology, University Clinical Centre of Kosovo, 10000 Prishtina, Kosovo; 4 Department of Preventive Cardiology and Lipidology, Medical University of Lodz, 93-338 Lodz, Poland

**Keywords:** congenital heart diseases, physical activity, exercise testing

## Abstract

The prolongation of the lifespan of patients with congenital heart diseases (CHD) has caused them to experience symptoms and complications not only related to the defect itself or late complications of surgical treatment but also to be at risk of population diseases such as obesity or ischemic heart disease. As recently as two decades ago, fewer than one in five CHD patients received formal advice on physical activity. Once a patient has been thoroughly evaluated and the risk has been stratified, the clinician can develop a detailed and individualized exercise prescription. This prescription is a formal plan that specifies the recommended frequency, intensity, duration, and type of physical activity. The goal is to maximize the health benefits of exercise while minimizing any potential risks. Although the anatomical classification of the defect and the previously implemented method of treatment allow the estimation of the possible late complications to a certain extent, in reality, the clinical condition may vary significantly in individual cases. For this reason, we prepared a practical approach regarding physical activity in patients with CHD based on hemodynamic and electrophysiological parameters, instead of focusing solely on specific defects. We also paid attention to special CHD patient populations in which separate indications regarding physical activity must be implemented.

## 1. Introduction

With the constant development of medicine and the much-needed improvement of care for children with congenital heart diseases (CHDs) in recent decades, survival to adulthood in this group has significantly increased [1,2,3,4,5]. Thus, in developed countries, treatment of patients with CHD is no longer the exclusive domain of pediatric cardiologists but also involves adult cardiology specialists [6]. Currently, this population consists mostly of adult patients [3]. The prolongation of the lifespan of patients with CHD has caused them to experience symptoms and complications not only related to the defect itself or late complications of surgical treatment but also to be at risk of population diseases such as obesity or ischemic heart disease [7,8]. The prevalence of CAD in ACHD patients is higher than in the general population [9]. Arterial hypertension is also more often diagnosed in CHD group, with the prevalence varying between 30 and 50% [8,10]. Obesity and overweight are at least as common amongst patients with congenital heart disease as in the general population [11,12]. On the brighter side, possibly due to health education, the proportion of ACHD patients who smoke has been reported to be smaller than in the general population [9,11].

It is well established that in the general population, to reduce the risk of developing these diseases, regular physical activity is recommended. This might be in stark contrast with traditional circumstances, due to which patients with CHD encounter parental and environmental overprotection, often resulting in reduced physical activity. As recently as two decades ago, fewer than one in five CHD patients received formal advice on physical activity [13]. Meanwhile, there has been emerging evidence regarding the benefits of physical exercise on cardiorespiratory fitness (CRF), prognosis, quality of life, and psychosocial health in patients with CHD [14]. The ESC and AHA guidelines strongly support the claim that the topic of physical activity should be discussed during each visit [5]. On the other hand, a vastly different anatomy and physiology of the heart of a CHD patient requires special attention and modification of existing recommendations created with the general population in mind [15]. Although the anatomical classification of the defect and the previously implemented method of treatment allow the estimation of possible late complications to a certain extent, in reality, the clinical condition may vary significantly in individual cases. For this reason, the current recommendations regarding physical activity in patients with CHD are based on hemodynamic and electrophysiological parameters, instead of focusing solely on specific defects [15].

## 2. Material and Methods

The aim of our study was to create a practical guide aiding clinicians in counseling adult congenital heart defect patients in terms of possible and desired levels of physical activity as this area of cardiologic care appears to be relatively uncovered.

An electronic search was completed in MEDLINE, SCOPUS, and Cochrane Database of Systematic Reviews according to existing guidelines pertaining to the topic. Recommendations were extracted and assembled from the identified articles to formulate a set of suggestions.

Databases were searched by using the following keywords: congenital heart diseases; congenital heart defect; exercise prescription; training program; physical activity.

Results were limited to the articles published within the 2010–2025 timeframe to capture up-to-date information regarding the subject.

## 3. Before Recommendations Can Be Made—Patients’ Condition Assessment

### 3.1. At Rest—Basic Methods

#### 3.1.1. Echocardiography

Echocardiography is generally considered the first-line modality for cardiovascular imaging in adults with CHD. Oftentimes, it is a sufficient diagnostic tool not only for reliable evaluation of cardiac morphology but also function and hemodynamic relevance of the malformations.

Echocardiographic assessment should follow the published general guidelines [16,17] but needs to take into account the specifics of ventricular pathophysiology in certain CHDs such as the central role of the right ventricle (RV) or the presence of intracardiac or extracardiac shunts [18]. The most important parameters that require assessment are:

- Ventricle systolic function: left ventricle ejection fraction (LVEF), tricuspid annular plane systolic excursion (TAPSE), and fractional area change (FAC) for RV function, 3D echocardiography when feasible;
- LV/RV pressure load: left/right ventricular outflow tract (LVOT/RVOT) peak systolic velocity to assess the LVOT/RVOT gradient;
- LV/RV volume load: the presence of valve regurgitation or shunt, the presence of RV or LV dilatation (LV end diastolic volume—LV EDV, RV end diastolic area—RV EDA)
- Pulmonary pressure estimation; in most risk stratification scale tricuspid valve regurgitation velocity (TRV) was used; however, in certain clinical scenarios (e.g., excentric jet) where reliable TRV measurement is not possible, other parameters, such as acceleration time of the pulmonary valve or mid-systolic notching, early diastolic pulmonary regurgitation velocity, or pulmonary artery dilatation, should be taken into consideration. In particularly complicated cases, right heart catheterization should be considered a golden standard.
- Aortic diameters: the aortic root diameter, measured in the parasternal long axis view (as the short axis may underestimate the diameter due to possible plane obliquity); the ascending aorta diameter, measured in end-diastole in the parasternal long axis view, often 1–2 intercostal spaces up [15,19,20].

#### 3.1.2. Arrythmia Detection: Resting ECG and 24-h (or Prolonged) ECG Monitoring

In CHD, arrhythmogenic substrates often arise from surgical scars, chamber dilation, pressure or volume overload, and long-standing myocardial remodeling rather than from acquired coronary or degenerative conduction system disease [21]. Atrial arrhythmias, such as intra-atrial reentrant tachycardia or atypical atrial flutter, are particularly prevalent in patients after atrial switch procedures or complex repairs involving extensive atriotomy. Ventricular arrhythmias may develop due to patch borders, fibrotic tissue, or residual outflow tract obstruction, as seen in repaired tetralogy of Fallot [22]. Moreover, conduction disturbances, including sinus node dysfunction and atrioventricular block, are common sequelae of surgical interventions or chronic hemodynamic stress. Unlike in the general population, where age-related fibrosis and ischemic injury predominate, arrhythmias in CHD often emerge at a younger age and may coexist with cyanosis, ventricular dysfunction, or pulmonary hypertension, significantly impacting exercise tolerance and prognosis [5]. A resting ECG can unveil conduction abnormalities, chamber enlargement, repolarization disturbances or accessory pathways, each of which may represent an arrhythmogenic substrate that could be unmasked or exacerbated by exertion [19,20]. Extended monitoring further allows detection of both symptomatic and silent supraventricular or ventricular arrhythmias. The assessment should be mostly focused on the prevalence of premature ventricular complex (PVC), coupled PVC, atrial fibrillation/atrial flutter, either non-sustained or sustained ventricular tachycardia [23]. Less than 500 PVC/24 h is considered clinically irrelevant [15]. In the case of arrythmia recognition, it is crucial to assess whether it worsens with exertion. When this initial testing fails to detect any significant abnormalities and there is a clinical suspicion of the arrythmia, further investigations, such as electrophysiology study or provocative tests, may be required. If the treatment is established and the clinical improvement is observed, re-assessment should be made to adjust the exercise prescription to the patient’s current condition.

#### 3.1.3. Assessment of Oxygen Saturation

Central cyanosis is largely excluded when transcutaneous saturations are higher than 95% at rest and during exercise. In patients with a potential right-to-left shunt, transcutaneous arterial saturation at rest should be recorded. Low arterial saturation due to pulmonary disease must not be overlooked [15].

### 3.2. Exercise Testing—Cardiopulmonary Exercise Test (CPET)

CPET enables objective measurement of the exercise capacity, which might be overestimated when self-reported by patients, may unmask impairments and arrhythmias even in asymptomatic patients, and is an integral part of the decision-making process [24]. CPET accompanied by 12-lead ECG is an important tool for assessing the baseline fitness of individuals and helps to choose the proper types of sport and level of intensity [25].

Most clinically valuable parameters to be assessed:

Cardiopulmonary indices:

Peak-oxygen consumption (peak VO_2_)—most studied CPET variable in CHD [24] and is one of the best predictors of morbidity and mortality in patients with CHD [26,27]. The prognostic cut-off value in patients with CHD is considered ≤36% of predicted peak VO_2_ [27]. However, serial decline may be more predictive than a single abnormal value [28]. Moreover, it is considered the most widely used measure of CRF. The spectrum of expected peak VO_2_ values differs depending on the ailment [27,28,29].

Heart rate reserve (HRR)—prognostically significant in CHD patients, particularly in the presence of cyanosis. Low HRR is an independent predictor of adverse outcomes. Chronotropic incompetence is very common in CHD patients. HRR is a strong predictor of mortality, transplant risk, and hospitalization in patients with Fontann circulation [25].

Ventilatory efficiency VE/VCO_2_ slope—useful parameter in the context of sub-maximal testing; often increased above the norm in patients with reduced LVEF, ventilation/perfusion mismatch, or right-to-left shunting. The prognostic cut-off value, predicting poor outcome in patients with CHD is >39 [27].

Ventilatory anaerobic threshold (VAT)—the most widely used measure of fitness during submaximal effort test, often decreased in CHD or deconditioning; according to the current guidelines distinction between the first and second ventilatory threshold (VT1 and VT2), it is recommended. VAT represents the same exertion stage as VT1 and marks the transition from purely aerobic to mixed aerobic–anaerobic metabolism. It also reflects the onset of lactate accumulation and bicarbonate buffering, which leads to a mild compensatory increase in ventilation [30,31].

Second ventilatory threshold (VT2, also termed as the anaerobic threshold) marks the intensity at which ventilation increases disproportionately relative to both VO_2_ and VCO_2_. This occurs when lactic acid production exceeds the buffering capacity of bicarbonate, causing metabolic acidosis. VT_2_ represents the upper limit of sustainable exercise intensity, above which steady-state metabolism is no longer possible, and fatigue ensues rapidly [30,31].

O_2_ pulse—decreased when impaired stroke volume or abnormalities in oxygen extraction is observed.

Arrhythmias—detection of arrhythmias during exercise increases the risk of sudden death by 6.6-fold [32].

Heart rate—measured continuously during exercise may reveal chronotropic incompetence, which in CHD patients, is often a symptom of ventricular dysfunction or ischemia [33].

Blood pressure—A normal blood pressure response during exercise includes a rise in systolic blood pressure by ≥25 mmHg, to a maximum of 220 mmHg (men) and 200 mmHg (women). An impaired response or a drop of systolic blood pressure during exercise should raise concern and be further diagnosed. The most common CPET abnormalities characteristic of different CHD phenotypes are summarized in Table 1. 

### 3.3. Additional Assessment Methods—To Be Used in Specific Circumstances

#### 3.3.1. Cardiovascular Magnetic Resonance

Cardiovascular magnetic resonance (CMR) may be required to assess right and left ventricular (LV) volumes and function, myocardial scars (which may act as a surrogate for arrhythmia risk), regurgitant fraction, visualization of prosthetic materials (conduits), and detailed morphological studies (e.g., pulmonary veins, coronary arteries), which can all impact on ventricular function in patients with CHD [19].

Borderline or pathological aortic diameter values found in echocardiography require additional cross-sectional imaging by CT or CMR.

#### 3.3.2. Computed Tomography

Computed tomography (CT) is the imaging modality of choice for the delineation of small anatomical structures such as coronary arteries and collateral arteries and for imaging parenchymal lung pathology [19].

Borderline or pathological aortic diameter values found in echocardiography require additional cross-sectional imaging by CT or CMR.

#### 3.3.3. Right Heart Catheterization

RHC provides precise measurements of intracardiac pressures, cardiac output, pulmonary and systemic vascular resistance, and oxygen saturations [5]. In CHD patients, RHC is particularly valuable for evaluating the severity and reversibility of pulmonary hypertension, quantifying intracardiac shunts, and assessing the hemodynamic impact of residual or complex lesions such as conduit stenosis, intracardiac shunts, or single-ventricle physiology [34]. In everyday clinical practice, RHC is usually recommended when the results of noninvasive diagnostic methods are inconclusive or not sufficient, such as in patients with a high echocardiographic probability of pulmonary hypertension, especially when considering limiting the practice of some or all sports, including competitive sports [19]. One has to keep in mind that RHC in CHD patients requires tailored protocols and experienced operators due to the heterogeneous anatomy, previous surgical repairs, and frequent presence of collateral vessels or baffles [35].

#### 3.3.4. Spirometry

Evaluation with spirometry should be included in patients with reduced arterial saturation without a cardiovascular explanation [15,19].

#### 3.3.5. Implantable Loop Recorder and an Electrophysiology Study

In patients with a high risk of arrythmia, especially in competitive athletes, additional methods of arrythmia assessment may be required before the final decision regarding physical activity is made [19].

#### 3.3.6. Coronary Artery Disease Diagnostic Methods–Imaging, Exercise Test and Coronarography

In the case of coronary artery disease suspicion, the diagnostic path for the general population is usually recommended. It is worth mentioning that even significant coronary artery disease can be symptomless in patients with a congenital heart defect, who underwent surgical treatment due to a possible partial denervation as a consequence of the procedure [36]. For this reason, a routine exercise test should be considered before an exercise prescription can be made. In the case of the signs of inducible myocardial ischemia, the invasive coronarography might be necessary to assess the risk and indication for revascularization [20]. The special high-risk group of premature coronary artery disease includes patients with coarctation of the aorta (CoA) and patients after coronary replacement, e.g., surgically treated transposition of the great arteries [36]. From a clinical viewpoint, it is essential to postpone physical activity recommendations until non-invasive coronary artery disease diagnostics are performed. In the case of an anomalous origin of coronary artery disease, imaging (invasive or noninvasive angiography) is used to rule out the malignant anatomical type of the anomaly, and exercise testing is recommended. In the case of high-risk anatomical conditions (e.g., acute angled take-off from the aorta resulting in a slit-like orifice with reduced lumen and anomalous coursing between the aorta and the pulmonary artery) or exercise-induced ischemia, only low-intensity skill sports may be allowed prior to surgical correction [20].

## 4. Physical Activity Prescription

**Figure 1:**
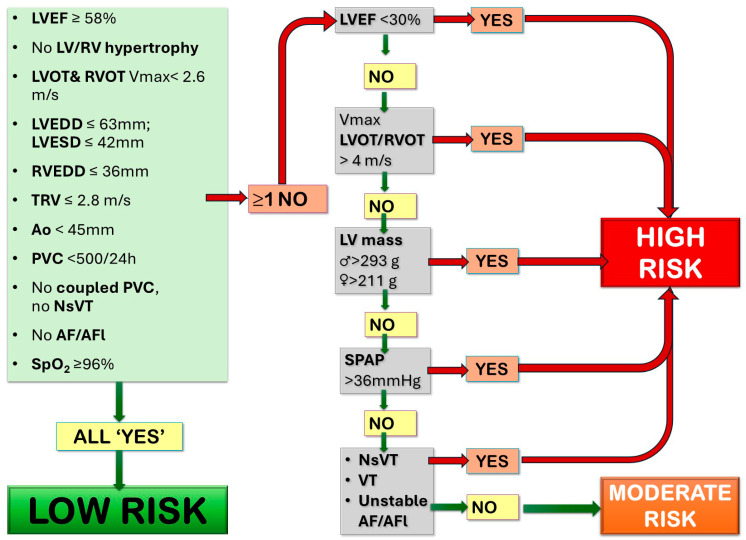
A flowchart helping to determine the recommended exercise level for patients with congenital heart disease. On the basis of Budts et al., Eur Heart J 2013 [15]. AF—atrial fibrillation; AFL—atrial fluttering; Ao—aorta; LV—left ventricle; LVEDD—left ventricular end diastolic diameter; LVESD—left ventricular end systolic diameter; LVEF—left ventricular ejection fraction; LVOT—left ventricular outflow tract; NsVT—non-sustained ventricular tachycardia; PVC-RV—right ventricle; RVEDD—right ventricular end diastolic diameter; RVOT—right ventricular outflow tract; SpO_2_—oxygen saturation; SPAP—systolic pulmonary artery pressure; TVRV—tricuspid regurgitation velocity; VT—ventricular tachycardia.

The process of risk profiling involves integrating the findings from the medical history, physical examination, cardiac imaging, and, most importantly, the objective exercise test. This synthesis allows the clinician to classify the patient into a general risk category—typically low, moderate, or high. A critical function of the risk stratification process is to identify absolute and relative contraindications to certain types of physical activity. The presence of specific “red flag” conditions necessitates significant activity restriction and, in some cases, may preclude participation in all but the lowest intensity activities [37]. The specific parameters determining the risk category classification are presented in Figure 1.

Once a patient has been thoroughly evaluated and the risk has been stratified, the clinician can develop a detailed and individualized exercise prescription. This prescription is a formal plan that specifies the recommended frequency, intensity, duration, and type of physical activity. The goal is to maximize the health benefits of exercise while minimizing any potential risks. The FITT-VP principle (Frequency, Intensity, Time, Type, Volume, and Progression) provides a comprehensive framework for structuring this prescription [38]. The main recommendations, taking into account the established level of risk in the CHD patients, in accordance with FITT-VP principles, are presented in Table 2.

### 4.1. Types of Physical Effort Activities

#### 4.1.1. Aerobic Training

Most studies concerning patients with congenital heart disease have focused on aerobic (endurance) exercises training (ET). Aerobic training (e.g., walking, cycling, swimming) primarily increases the cardiac output via augmented stroke volume and heart rate, improving peak VO_2_, endothelial function, insulin sensitivity, and overall quality of life [20].

The minimum threshold of aerobic exercise intensity for patients with congenital heart disease (CHD) remains insufficiently defined, and absolute intensity prescriptions differ considerably depending on the underlying defect and the individual’s baseline CRF. Exercise programs frequently determine aerobic training intensity from maximal heart rate (HRmax) obtained during exercise testing. Most interventions apply workloads within 60–80% of HRmax [41,42,43]; however, studies have also reported effective protocols at intensities as low as 40% of HRmax [44] and as high as 95% of HRmax, the latter typically within interval training regimens.

Recent findings indicate that high-intensity interval training (HIIT) may safely enhance CRF in adults with congenital heart disease (CHD). A systematic review encompassing 3 studies and 87 adults found no serious adverse events and noted improvements in peak oxygen uptake among participants undergoing HIIT regimens [45]. Additional data from a randomized controlled trial in adults with the corrected tetralogy of Fallot demonstrated that interval training significantly improved VO_2_-peak, vascular endothelial function, and biomarkers like NT-proBNP and fibrinogen [46]. Although randomized studies remain limited, this emerging evidence supports HIIT as a promising, lesion-specific intervention in CHD rehabilitation.

#### 4.1.2. Resistance Training

Resistance training (e.g., machine or free-weight exercises, use of bands) produces a brief, higher afterload with less sustained tachycardia, which increases muscular strength, bone density, and functional capacity; when performed at light to moderate loads with avoidance of prolonged Valsalva, it has favorable hemodynamic profiles for many CHD patients.

The systematic review by Hao et al. showed a moderate improvement in muscle strength achieved with strength training alone, with a minor improvement in peak VO_2_ in a group of patients with congenital heart disease. The most effective method for VO_2_-peak improvement was combined strength training with inspiratory muscle training [47].

Rest intervals of approximately 60 s between sets are advised to permit normalization of blood pressure and heart rate. Resistance training should be initiated at low intensities and gradually progressed to reach target levels, with movements performed at a controlled tempo of 2/0/2 s (eccentric/isometric/concentric) [42]. To elicit clinically relevant muscle hypertrophy, a frequency of 2–3 non-consecutive sessions per week is generally required [48]. Exercise prescriptions should emphasize large functional muscle groups of both the upper and lower extremities, with particular attention to the lower limbs in individuals with Fontan physiology, where enhanced skeletal muscle pump activity may provide additional circulatory support [49,50].

### 4.2. How to Assess the Recommended Exercise Intensity?

Contemporary literature favors an individual approach and recommendations based on relative exercise intensity. For this purpose, the measured objective CPET values with particular emphasis on the maximal heart rate is essential. It was also confirmed that objective CPET parameters corelate well with subjective estimates such as the rate of perceived exertion (RPE).

Up-to-date position statement suggests determining the exercise intensity by assessing VT1 and VT2. Typically, ‘ET zones’ could then be established as low-intense (at an HR or workload below VT1)—a level which should not be exceeded in high-risk patient groups; moderate–intense (at an HR of workload between VT1 and VT2)—appropriate for moderate-risk groups; and high-intense (at an HR or workload above VT2)—which may be considered in low-risk groups. If VT cannot be traced due to technical constraints, clinicians may recommend the exercise intensity on the basis of the maximal heart rate (HRmax) achieved during CPET (low intense: <55% of HRmax, moderate intense: 55–74% of HRmax; high intense: 75% of HRmax or more) [30,31].

The RPE Borg scale is based on the patient’s subjective sensations during a workout. Recommendations divide exercise load into three categories: low intensity (RPE Borg Scale 11–12)—light to moderate subjective description of exercise intensity with individual feelings of not exceeding light perspiring; moderate intensity—(RPE Borg Scale 13–14)—moderate to hard subjective description of exercise intensity with individual feelings of intensive perspiring to light sweating; high intensity—(RPE Borg Scale 15–17)—hard to very hard subjective description of exercise intensity with individual feelings of sweating and hard working [15].

Subjective assessment with the use of the Borg scale may be especially useful in CHD patients, in whom chronotropic incompetence or atrial fibrillation may be present and affecting the practical usefulness of a fixed percentage MHR application. In this group of patients, the basic estimation methods, like ‘if you can talk while exercising’ rule to approximate moderate relative exercise intensity, may prove helpful in everyday practice [51].

Recent advances in digital health have introduced new opportunities for remote and home-based management, which might prove particularly suitable for patients with CHD. Remote monitoring technologies, including wearable sensors and implantable cardiac devices, enable the continuous assessment of physical activity levels, arrhythmic events, and overall adherence to exercise prescription [52].

## 5. Special Needs Patient Population

### 5.1. Pulmonary Hypertension

In patients with pulmonary arterial hypertension (PAH) secondary to congenital heart disease (CHD), right ventricular (RV) afterload is chronically elevated. Initially, the right heart undergoes adaptive remodeling characterized by concentric hypertrophy to preserve systolic function. However, this compensatory mechanism may become maladaptive over time, resulting in progressive systolic dysfunction and RV dilation. In individuals with Eisenmenger syndrome, this adaptive hypertrophic response may remain sustained into late adulthood.

In the previously implemented clinical practice, ET and cardiopulmonary rehabilitation were considered contraindicated in patients with pulmonary hypertension (PH), owing to concerns regarding patient safety, including the potential risk of sudden cardiac death, aggravation of pulmonary vascular remodeling, and progressive right ventricular dysfunction. Accordingly, patients with PH were routinely advised to abstain from physical activity.

While low-to moderate-intensity physical activity is generally well tolerated in patients with PH and has been shown to confer clinical benefits, high-intensity exercise may carry an elevated risk of adverse events, including hypotension due to reduced cardiac output, syncope, and sudden cardiac death. However, a meta-analysis by Zeng et al. reported a very low incidence of adverse events (~3.5%), with no major complications such as severe right heart failure, disease worsening, or death [53].

A meta-analysis of 9 randomized controlled trials (total number of 302 participants) demonstrated that ET significantly improved VO_2_-peak (mean increase of approx. +2.79 mL·kg^−1^·min^−1^), anaerobic threshold (approx. +107 mL·min^−1^), and 6-min walk distance (6MWD) by an average of +46.7 m. In addition, both physical and mental scores in the SF-36 quality of life questionnaire improved significantly [54]. Systematic reviews confirm that physical training can reduce resting pulmonary artery systolic pressure (PASP), improve pulmonary perfusion, and enhance overall cardiopulmonary function [53]. Although it is relatively rare, some patients with PAH have exertional angina, resulting from compression of the left main coronary artery by a dilated pulmonary artery, such a phenomenon may be exacerbated during physical activity.

### 5.2. Desaturation

In individuals with cyanotic congenital heart disease (CHD), baseline arterial oxygen saturation may be markedly reduced, with significant desaturation occurring even during low-intensity activity. Those with right-to-left shunting or diminished pulmonary blood flow are particularly susceptible to desaturation during exercise. For patients with severe cyanosis, both exercise intensity and duration should therefore be guided by symptom limitation and subjective ratings of perceived exertion. In selected frail patients, a modest improvement in oxygen saturation may be achieved through supplemental oxygen, which can occasionally be considered as an adjunct during exercise testing or training [42].

### 5.3. Pathologies of the Aorta

Following the repair of a coarctation, the primary long-term concern is systemic hypertension. Patients may present with normotension at rest yet demonstrate a pronounced hypertensive response even to minimal physical exertion, underscoring the importance of careful monitoring of blood pressure and heart rate during exercise [29,42,55]. In individuals with repaired coarctation of the aorta, exercise testing is recommended to evaluate the hemodynamic response to physical stress. Based on the results, individuals with a significant hypertensive response to exercise may need to avoid activities with a high static load that can cause a sharp rise in blood pressure. Regular aerobic exercise is generally beneficial and encouraged to help manage resting blood pressure [55,56]. Patients presenting with significant aortic dilatation or obstructive lesions should be restricted to low-intensity ET until definitive surgical repair has been performed and formal medical clearance obtained [42]. Engagement in moderate- to high-intensity aerobic or resistance ET is theoretically associated with an increased risk of promoting aortic dilatation or, in rare instances, precipitating aortic dissection. Consequently, such forms of training are contraindicated in the presence of moderate or greater degrees of aortic dilatation, as well as in cases of documented aortic aneurysm or pseudoaneurysm [57].

### 5.4. Cardiac Implantable Electronic Devices

Following implantation, excessive upper extremity activity should be restricted for approximately 3–4 weeks to reduce the risk of lead displacement. In the longer term, patients with cardiac implantable devices are generally advised to avoid high-impact physical activities that may compromise device integrity. Endurance-based sports involving repetitive arm movements, such as long-distance swimming, can place mechanical stress on transvenous leads and may cause a predisposition to lead damage; such activities should therefore be approached with caution [42,58].

In individuals with ICDs, exercise intensity should be generally prescribed 10–15 beats per minute below the programmed detection threshold to minimize the risk of inappropriate shocks, unless advanced arrhythmia discrimination features are confirmed [59,60]. In patients with pacemakers, exercise generally poses fewer restrictions; however one should keep in mind that heart rate may be an unreliable marker of training intensity. Furthermore, individuals with complete heart block who rely on atrial-sensed, ventricular-paced modes may reach the device’s upper tracking limit at higher exercise intensities, resulting in the abrupt loss of atrioventricular synchrony [42].

### 5.5. Fontan Circulation

Fontan circulation is characterized by the absence of a sub-pulmonary ventricle, resulting in passive pulmonary blood flow and inherently limited hemodynamic reserve. Such altered physiology constrains the ability to augment cardiac output during exertion, leading to reduced exercise tolerance [61]. Consequently, many Fontan patients adopt a sedentary lifestyle, which exacerbates skeletal muscle atrophy and further diminishes functional capacity [62]. According to European Society of Cardiology guidelines, moderate symptom-limited aerobic exercise is to be recommended to improve muscular strength and quality of life [5] A randomized trial combining aerobic and inspiratory muscle training increased peak VO_2_ by ~23% (27.0→33.3 mL·kg^−1^·min^−1^) versus usual care, with additional benefits from inspiratory training alone and no training-related serious events [62,63]. Multiple systematic reviews and meta-analyses confirm consistent improvements in exercise capacity, with signals for better ventilatory efficiency and quality of life, and very low adverse-event rates [64]. Leg-focused resistance programs have also proved beneficial for Fontan patients, as targeted strengthening of the lower limbs augments the skeletal muscle pump and venous return in the absence of a subpulmonary ventricle [50,65].

## 6. Conclusions

The clinical approach to physical activity in patients with congenital heart disease (CHD) has undergone a profound transformation over the past several decades and can be summarized in the following bullet points:

1. For the vast majority of patients, physical activity is not only safe but essential and should be strongly promoted.
2. Activity restrictions are now reserved for a very small subset of patients with specific high-risk conditions.
3. The contemporary role of the healthcare professional has expanded beyond simply permitting activity.
4. Physical activity counseling is considered an essential component of every patient interaction, regardless of whether the patient’s clinical status warrants any restrictions.
5. The management of physical activity is a dynamic, lifelong process. The initial prescription is not static but must be subject to ongoing surveillance and adjustment

Historically, clinical practice was dominated by a paradigm of restriction, where patients were frequently advised to limit their physical activity. The overwhelming consensus is that a sedentary lifestyle poses a greater threat to the long-term health of most CHD patients than does participation in appropriate physical activity. The clinical imperative is therefore to safely guide patients toward achieving and maintaining an active life. It now involves actively counteracting the ingrained fear, parental overprotection, and physical deconditioning that resulted from previous medical guidance. Regular follow-up, including serial exercise testing, is essential for monitoring a patient’s clinical trajectory. A spontaneous decline in a patient’s habitual activity level should be treated as a potential early warning sign of clinical deterioration, prompting a thorough re-evaluation. Through a continuous cycle of evaluation, risk stratification, and prescription—all conducted within a framework of shared decision-making and empowering patient counseling—healthcare professionals can effectively and safely integrate physical activity as a core therapeutic modality, helping patients with congenital heart disease live longer, healthier, and more fulfilling lives.

## Figures and Tables

**Table 1:** Main CPET parameters abnormalities in different CHD phenotypes. Adapted and simplified from Guglielmi G et al., 2025 [25].

CHD Type	Peak VO_2_	VE/VCO_2_ Slope	VAT	HRR
**Biventricular circulation** **with systemic LV**	~71% predicted;	Normal/mildly elevated	Often reduced	Frequently reduced.
**Biventricular circulation** **with systemic RV**	~63–67% predicted	Mildly elevated	Reduced	Frequently reduced;
**Fontan circulation**	~59% predicted	Mildly elevated	Reduced	Frequently reduced
**Eisenmenger syndrome**	~42% predicted	Significantly elevated	Markedly reduced	Frequently reduced

VAT—ventilatory anaerobic threshold; CHD—congenital heart disease; HRR—heart rate reserve production; LV—left ventricle; RV—right ventricle; VE/VCO_2_—ventilation to carbon dioxide output; VO_2_—oxygen uptake.

**Table 2:** FITT-VP principle application for different CHD risk categories [20,38,39,40]. RPE—rate of perceived exertion; VAT—ventilatory anaerobic threshold; VT1—first ventilatory threshold.

FITT-VP Component	Low Risk	Moderate Risk	High Risk (Medically Supervised)
**Frequency**	5–7 days/week	3–5 days/week	3–5 days/week
**Intensity (Aerobic)**	Moderate to vigorous. RPE 13–17. Can progress to activity above VAT/VT1 or even VT2.	Low to Moderate. RPE 11–14. Generally maintain activity between VAT/VT1 and VT2.	Low. RPE 9–11. Activity must remain below VAT/VT1. Continuous monitoring may be required.
**Time (Duration)**	30–60 min per session.	20–40 min per session. May start with 10–15 min bouts.	15–30 min per session. Start with short bouts (5–10 min) with frequent rest periods.
**Type**	Wide variety of aerobic and recreational sports. Competitive sports often permissible after evaluation.	Rhythmic, large muscle group activities (e.g., walking, cycling, swimming). Avoid high-intensity competitive sports.	Low-impact, low-intensity activities (e.g., slow walking, light cycling, water aerobics).
**Volume**	Aim for ≥150 min moderate or ≥75 min vigorous activity/week.	Gradually work toward 150 min moderate activity/week.	Volume is secondary to safety; focus on consistency and gradual progression of duration at low intensity.
**Progression**	Gradual increase in intensity and duration as tolerated.	Slow and gradual progression. Increase duration before intensity. Re-evaluate before progressing to vigorous activity.	Very slow progression under medical guidance. Any change requires re-evaluation.
**Resistance Training**	2–3 days/week. Moderate intensity.	2 days/week. Low intensity, higher repetitions (12–15). Avoid Valsalva maneuver.	1 to 2 days/week. Very low intensity, focusing on activities of daily living. May require supervision.

## Abbreviations

The following abbreviations are used in this manuscript:

6MWT	6-min walk test
AF	Atrial fibrillation
AFl	Atrial fluttering
Ao	Aorta
ccTGA	Congenitally corrected transposition of the great arteries
CHD	Congenital heart diseases
CMR	Cardiovascular magnetic resonance
CPET	Cardiopulmonary exercise test
CRF	Cardiorespiratory fitness
CT	Computed tomography
ECG	Electrocardiogram
EDA	End diastolic area
EDV	End diastolic volume
ET	Exercise training
FAC	Fractional area change
HITT	High-intensity interval training
HRR	Heart rate reserve
LV	Left ventricle
LVEDD	Left ventricular end diastolic diameter
LVEF	Left ventricular ejection fraction
LVESD	Left ventricular end systolic diameter
LVOT	Left ventricular outflow tract
NsVT	Non-sustained ventricular tachycardia
PAH	Pulmonary arterial hypertension
PASP	Pulmonary artery systolic pressure
PVC	Premature ventricular complex
RPE	Rate of perceived exertion
RV	Right ventricle
RVEDD	Right ventricular end diastolic diameter
RVOT	Right ventricular outflow tract
SPAP	Systolic pulmonary artery pressure
SpO_2_	Oxygen saturation
TAPSE	Tricuspid annular plane systolic excursion
TRV	Tricuspid regurgitant velocity
VAT	Ventilatory anaerobic threshold
VE/VCO_2_	Ventilation to carbon dioxide output
VO_2_	Oxygen uptake
VT	Ventricular tachycardia
